# Ammoxetine attenuates diabetic neuropathic pain through inhibiting microglial activation and neuroinflammation in the spinal cord

**DOI:** 10.1186/s12974-018-1216-3

**Published:** 2018-06-07

**Authors:** Ting-Ting Zhang, Rui Xue, Shi-Yong Fan, Qiong-Yin Fan, Lei An, Juan Li, Lei Zhu, Yu-Hua Ran, Li-Ming Zhang, Bo-Hua Zhong, Yun-Feng Li, Cai-Ying Ye, You-Zhi Zhang

**Affiliations:** 1 Institute of Pharmacology and Toxicology, Beijing Key laboratory of Neuropsychopharmacology, 27th Taiping Road, Haidian District, Beijing, 100850 China; 2 Department of Pharmacology, Institute of Basic Medical Sciences, Chinese Academy of Medical Sciences and Peking Union Medical College, Beijing, 100005 China; 3 Beijing Laboratory for Food Quality and Safety, Beijing Technology and Business University (BTBU), No.11, Fucheng Road, Haidian District, Beijing, 100048 China

**Keywords:** Ammoxetine, SNRI, Diabetic neuropathic pain, Glia, Cytokines, Mitogen-activated protein kinase

## Abstract

**Background:**

Diabetic neuropathic pain (DNP) is a common and distressing complication in patients with diabetes, and the underlying mechanism remains unclear. Tricyclic antidepressants (TCAs) and serotonin and norepinephrine reuptake inhibitors (SNRIs) are recommended as first-line drugs for DNP. Ammoxetine is a novel and potent SNRI that exhibited a strong analgesic effect on models of neuropathic pain, fibromyalgia-related pain, and inflammatory pain in our primary study. The present study was undertaken to investigate the chronic treatment properties of ammoxetine on DNP and the underlying mechanisms for its effects.

**Methods:**

The rat model of DNP was established by a single streptozocin (STZ) injection (60 mg/kg). Two weeks after STZ injection, the DNP rats were treated with ammoxetine (2.5, 5, and 10 mg/kg/day) for 4 weeks. The mechanical allodynia and locomotor activity were assayed to evaluate the therapeutic effect of ammoxetine. In mechanism study, the activation of microglia, astrocytes, the protein levels of pro-inflammatory cytokines, the mitogen-activated protein kinases (MAPK), and NF-κB were evaluated. Also, microglia culture was used to assess the direct effects of ammoxetine on microglial activation and the signal transduction mechanism.

**Results:**

Treatment with ammoxetine for 4 weeks significantly relieved the mechanical allodynia and ameliorated depressive-like behavior in DNP rats. In addition, DNP rats displayed increased activation of microglia in the spinal cord, but not astrocytes. Ammoxetine reduced the microglial activation, accumulation of pro-inflammatory cytokines, and activation of p38 and c-Jun N-terminal kinase (JNK) in the spinal cord of DNP rats. Furthermore, ammoxetine displayed anti-inflammatory effects upon challenge with LPS in BV-2 microglia cells.

**Conclusion:**

Our results suggest that ammoxetine may be an effective treatment for relieving DNP symptoms. Moreover, a reduction in microglial activation and pro-inflammatory release by inhibiting the p-p38 and p-JNK pathways is involved in the mechanism.

## Background

Diabetic neuropathic pain (DNP) represents a distressing complication that develops in approximately 20% of all patients with diabetes, manifesting as spontaneous pain, paresthesia, dysethesia, hyperalgesia, and allodynia [1]. In addition, the constant pain is often associated with sleep disturbances, anxiety, and depression that collectively decrease the life quality of patients with diabetes [2]. The treatment of DNP is challenging due to the unclear pathophysiology. Antidepressants, including tricyclic antidepressants (TCAs) and serotonin and norepinephrine reuptake inhibitors (SNRIs), are recommended as first-line treatments for DNP [3, 4]. Among these drugs, duloxetine is more preferred and firstly approved for DNP by the US Food and Drug Administration (FDA) [5]. These antidepressants provide multiple benefits by simultaneously reducing pain and exerting mood-improving effects [6, 7]. However, the analgesic efficacies are still incomplete in the clinic. Furthermore, TCAs generally cause multiple nefarious side effects [8]. Duloxetine is disallowed for patients with liver disease, and anticonvulsants should be avoided in patients with edema [9–13]. Thus, more efficacious and safer agents for improved therapies are clearly needed.

The pain-relieving effects of antidepressants are conventionally attributed to their potentiation of the transmission of serotonin (5-HT) and norepinephrine (NE) signals in the descending pain-inhibitory pathways [14, 15]. However, increased 5-HT and NE levels in the spinal cord and rostral ventromedial medullary (RVM) neurons have been observed in diabetic rats [16, 17]. As 5-HT and NE also play facilitatory roles in descending pain modulating pathways, these changes probably account for the enhanced pain in patients with diabetic neuropathy [18, 19]. Therefore, the mechanism underlying the analgesic effects of SNRIs may not simply depend on the regulation of monoamine neurotransmitters.

Glial cells in the spinal cord are responsible for the development and maintenance of neuropathic pain. The contributions of spinal cord microglia and astrocytes to DNP have been investigated recently. According to the studies by Tsuda and Wodarski [20], spinal microglia, but not astrocytes, are activated in STZ-treated diabetic rats. Microglial activation is accompanied by the release of cytokines and phosphorylation of the mitogen-activated protein kinases (MAPKs) including p38-MAPK, extracellular signal-regulated protein kinase (ERK), and c-Jun N-terminal kinase (JNK), which are known to participate in the generation of pain hypersensitivity [21, 22]. However, reports on the involvement of glial cells in the analgesic effects of antidepressants on DNP are lacking.

**Fig. 1:**
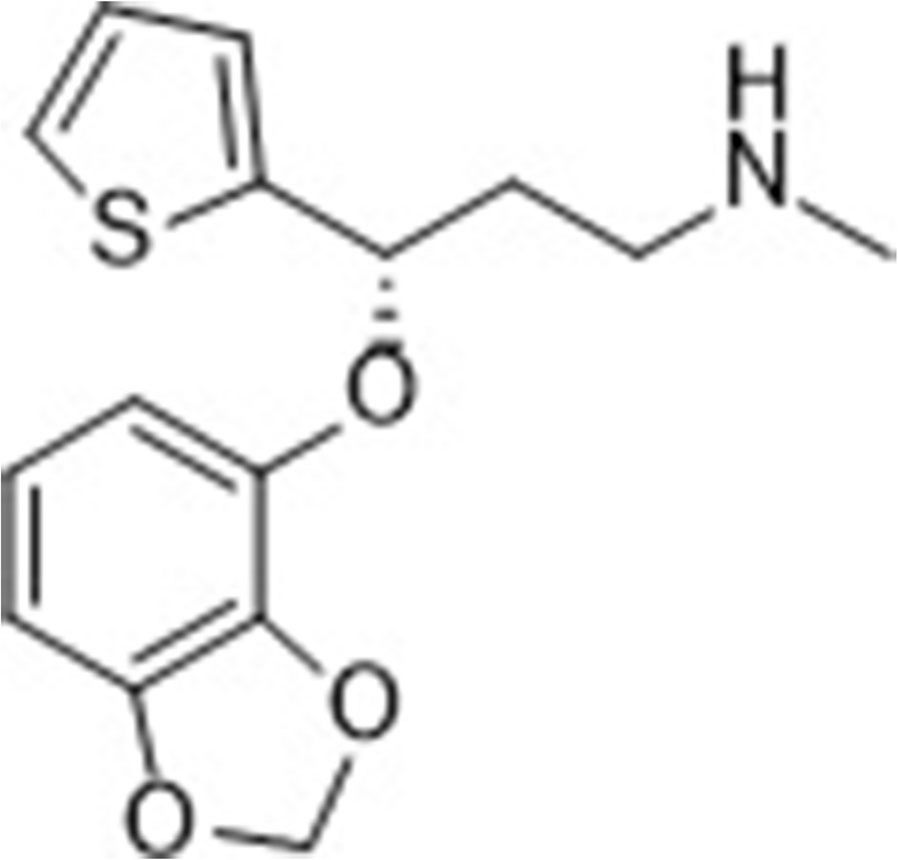
Chemical structure of ammoxetine

It has been reported that the hepatotoxicity of duloxetine might be due to the epoxidation of its naphthyl ring [23]. Ammoxetine is a candidate screened from a series of compounds that synthesized from reforming the naphthyl ring of duloxetine (Fig. 1). Our published data have proved that ammoxetine is a potent and balanced SNRI with superior antidepressant activity and lower cytotoxicity in HepG2 cells compared to duloxetine [24–26]. Ammoxetine displays acute efficacy in reversing mechanical allodynia in models of neuropathic pain, fibromyalgia-related pain, and inflammatory pain [27]. In this study, we evaluated the efficacy of a chronic ammoxetine treatment in DNP in STZ-induced diabetic rats. In addition, we also investigated the underlying mechanisms by focusing on the roles of spinal glia and cell signaling molecules (Fig. 1).

## Methods

### Animals

Rats were purchased from the Beijing Vital River Laboratory Animal Technology, Co., Ltd. (Beijing, China, permit number SCXK(JING) 2012-0001). All animals are group-housed under a 12-h light/dark cycle at room temperature (23 ± 1 °C) for 3–6 days. Animals had free access to food and water at all times before the experiments. The tests were conducted in the light cycle. The procedures on animal experimentation were approved by the Animal Care Committee of the Chinese People’s Liberation Army General Hospital (Beijing, China). The maintenance and handling of the rats were consistent with the guidelines of the National Institutes of Health, and adequate measures were taken to minimize animal discomfort.

### Drugs

Duloxetine hydrochloride was purchased from Shanghai Wandai Pharmaceutical Corporation, Ltd. (Shanghai, China). Ammoxetine was synthesized at our institute (Beijing Institute of Pharmacology and Toxicology). Streptozocin (STZ) was purchased from Sigma-Aldrich (St. Louis, MO, USA). Ammoxetine and duloxetine were dissolved in distilled water. Streptozocin was dissolved in 0.1 M citrate buffer (pH 4.2). Ammoxetine and duloxetine were administrated orally (p.o.) at a concentration of 1.0 ml/kg. The doses for each drug used in the experiment are discussed below.

### Induction of diabetic neuropathic pain and experimental protocols

Rats weighing 250–280 g were fasted overnight and then intraperitoneally (i.p.) injected with a single 60 mg/kg dose of freshly prepared STZ. The age-matched rats in the control group received equal volumes of the 0.1 M citrate buffer (pH 4.2). One week after the STZ injection, diabetes was confirmed by determining blood glucose levels in blood samples collected from tail veins using glucometer IME-DC (Bayer, Leverkusen, Germany). Rats with blood glucose levels ≥ 16 mM were considered diabetic and selected for the experiments. Diabetic rats with neuropathic pain were defined as the 50% PWT < 5 g measured by von Frey hair test [28].

Drugs were administrated 2 weeks after the STZ injection. Diabetic rats were treated with ammoxetine (2.5, 5 and 10 mg/kg, p.o.), duloxetine (10 mg/kg, p.o.), or distilled water once a day for 4 weeks to evaluate the efficacy of chronic ammoxetine treatment. Age-matched control rats were treated with distilled water. The mechanical allodynia thresholds were measured once a week until the end of the experiments. At the 2nd and 6th weeks after STZ injection, blood glucose levels were measured to confirm the maintenance of hyperglycemia in STZ-diabetic rats.

### Mechanical allodynia (von Frey hair test)

The mechanical allodynia of diabetic rats was assessed on the left hind paws using a method reported by Chaplan SR [29]. Briefly, rats were placed in test cages with a metal-mesh floor and were allowed to habituate for at least 30 min. Von Frey filaments (Touch-Test® Sensory Evaluators, NC12775-99, North Coast Medical Inc., San Jose, CA, USA) of 0.6, 1.0, 1.4, 2.0, 4.0, 6.0, 8.0, 10.0, 15.0, and 26.0 g were applied to the plantar surface of the left hind paw. A positive or negative response was defined as a paw withdrawal response from the pressure of a filament or the lack of a response within 6 s, respectively. Initially, a 2.0 g force filament was used for the diabetic rats, and a 6.0 g force filament was used for the control rats. If a positive response to a given filament was observed, the next smaller filament was then used. If a negative response occurred, the next larger filament was used. The test continued until four responses were collected after the first change in response. The tactile stimulus producing a 50% likelihood of paw withdrawal threshold (PWT) calculated using an adaptation of the Dixon up-down paradigm was used to calculate the nociceptive threshold.

### Open field test

Locomotor activity was measured using the open field text. Individual rats were placed in an arena (diameter 122 cm, height 45 cm) with a base that was equally divided into 16 sectors and allowed to move freely for 5 min. Subsequently, the numbers of times the rats crossed the grids and reared were counted [24].

### BV2 cell culture and activation

BV2 cells, which were purchased from Cell Bank of Chinese Academy of Medical Sciences, Beijing, China, were maintained in Dulbecco’ s Modified Eagle Medium (DMEM, Invitrogen, Camarillo, USA) with 10% fetal bovine serum (FBS, Invitrogen, Camarillo, USA) in a 5% CO2 incubator. Before incubated with LPS and ammoxetine, cells were seeded into 24-well plate at a density of 1 ✕ 10^5^ cells/ml with serum-free DMEM overnight. On the next day, cells were stimulated with LPS (200 ng/ml) in the absence or presence of the indicated concentrations of ammoxetine for 24 h and then collected for western blot and cell immunofluorescence analyze. The culture medium was collected for the analysis of tumor necrosis factor-α (TNF-α) release by ELISA.

### Cell viability assay

Cell viability was determined by the tetrazolium salt 3-[4,5-dimethylthiazol-2-yl]-2,5-diphenyltetrazolium bromide (MTT, Sigma-Aldrich, USA) assay. Cells were plated into 96-well culture plates at a density of 5 ✕ 10^4^ cells/ml with 200 ml culture medium per well. Following treatment with different concentrations (0.1, 1, 10, 20, 50, and 100 μM) of ammoxetine for 24 h, 5 mg/ml MTT solution was added to each well and incubated at 37 °C for 4 h. The medium was aspirated, and 200 ml dimethyl sulfoxide was added. The absorbance value was measured using a multi-well spectrophotometer (Perkin Elmer, USA) at 490 nm.

### Preparation of protein samples

Rats were euthanized immediately after the last pain behavior test. The spinal cords were promptly removed and immediately frozen and stored in liquid nitrogen until assay. Samples were weighed and homogenized in RIPA lysis buffer containing proteinase inhibitors and protein phosphatase inhibitors. After incubation for 30 min on ice, tissue homogenates were centrifuged at 12000 rpm for 30 min at 4 °C and the supernatant was then collected. Cells were washed with PBS and collected with RIPA buffer, and protein solution was collected after centrifugal. The protein concentration was determined using a BCA assay kit (Thermo, Rockford, USA).

### Western blot

Protein samples (40 μg) were subjected to electrophoresis on a 10% Tris-HCl SDS-PAGE gel (Bio-Rad, Hercules, CA) and transferred onto a PVDF membrane (Merck Millipore, MA, USA). After blocking nonspecific binding sites with 5% non-fat milk or bovine serum albumin in 0.1% Tween-20/Tris-buffered saline (TBS-T) for 1 h at room temperature, membranes were incubated with primary antibodies GFAP (Merck Millipore), iba-1 (Abcam), Total-p38, p-p38, total-ERK, p-ERK, total-JNK, p-JNK, p-ATF2, I-κBα, and nuclear factor-kB/P65 (NF-Κb/P65) (Cell Signaling Technology) overnight at 4 °C. Then, membranes were washed with TBS-T and probed with a secondary antibody (1:5000, Santa Cruz Biotechnology, CA, USA) for 1 h at room temperature. After washing, the protein bands were visualized using an enhanced chemiluminescence assay (Merck Millipore), according to the manufacturer’s instructions. The membranes were re-probed with an anti-β-actin antibody (Merck Millipore) after stripping. The signal intensity of the bound primary antibody was quantitatively analyzed with a Gel-Pro analyzer 4 and normalized to the loading control β-actin.

### ELISA

The contents of TNF-α, interleukin-1β (IL-1β), and interleukin-6 (IL-6) in the lumbar spinal cord homogenates and cell culture medium were measured using enzyme-linked immunosorbent assays (ELISAs; Invitrogen, Camarillo, CA). Briefly, 50 μl of each sample and the biotinylated anti-TNF-α, anti-IL-1β, or anti-IL-6 antibodies were added to two wells of the appropriate 96-well plate and incubated for approximately 2 h at room temperature. The plates were then washed and incubated with 100 μl of streptavidin-HRP for 30 min at room temperature. After incubation for 30 min, 100 μl of the stabilized chromogen were added to each well and 100 μl of stop solution were added to stop the reaction. The absorbance was detected at 450 nm. The concentrations of IL-1β, IL-6, and TNF-α were calculated according to the standard curve and presented as pg/mg protein in tissue homogenate or pg/ml in culture medium.

### Immunofluorescence

Rats were deeply anesthetized and the lumbar spinal cords were collected after transcardial perfusion with PBS followed by 4% paraformaldehyde (PFA). Tissue was then immersed in 4% PFA for 12 h following perfusion, cryoprotected in 30% sucrose, and embedded in OCT and frozen. Tissue was cryosectioned (10 μm), and sections were dried, washed three times in PBS, and blocked with 5% goat serum and in PBS for 1 h at room temperature. Primary antibodies were diluted in antibody buffer (containing 0.2% Triton X-100 and 5% goat serum) as follows: anti-iba1 (1:200, Wako), anti-GFAP (1:200, Millipore), and incubated overnight at 4 °C. After washing, samples were labeled with a secondary antibody conjugated Alexa647 (Abcam) and counter-stained with DAPI. Microglia cells were fixed in paraformaldehyde 4% for 20 min and then washed three times with PBS. Sections were blocked with 10% donkey serum and 0.3% Triton X-100 at room temperature for 30 min. Primary iba1 antibody (1:200, Wako) was incubated overnight at 4 °C. Corresponding anti-rabbit antibody conjugated Alexa647 (Abcam) was incubated at room temperature. Slides were imaged using Nikon A1 confocal laser microscope system and quantified using Image-Pro Plus 7.0 software.

### Statistical analysis

The statistical analysis was performed using GraphPad Prism software (GraphPad Prism 5.0, version 2.0; GraphPad Software Inc., San Diego, CA, USA). The data are all expressed as means ± SEM. The data obtained from the time-course measurements of mechanical thresholds and open field test behavior analyses were conducted using two-way repeated-measures ANOVA followed by Bonferroni’s post hoc analysis. ELISA and western blot data were analyzed using Student’s *t* test or a one-way analysis of variance (ANOVA) followed by Dunnett’s test. For data exhibiting unequal variances, a Mann-Whitney *U* test or a Kruskal-Wallis test followed by Dunn’s multiple comparison test were applied. Probability values of less than 0.05 were considered statistically significant.

## Results

### Ammoxetine relieved the mechanical allodynia in diabetic rats

**Fig. 2:**
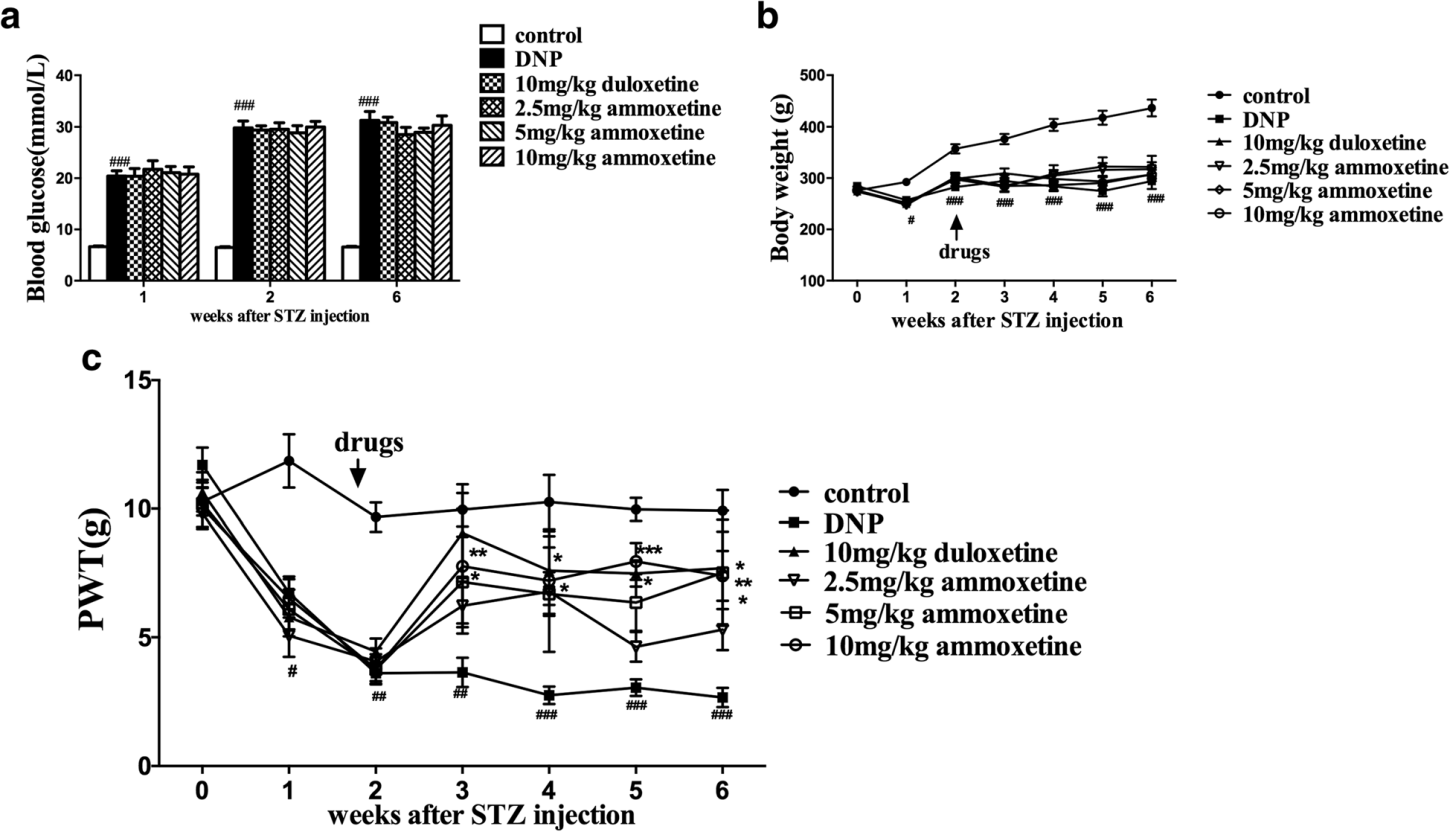
Effects of ammoxetine on mechanical allodynia in DNP rats. **a** Blood glucose levels in rats treated with STZ and vehicle. **b** Time course of body weight changes in rats treated with STZ and vehicle. **c** Ammoxetine treatments administered for consecutive 4 weeks increased the PWT in DNP rats (*n* = 8). ^#^*P* < 0.05, ^##^*P* < 0.05, and ^###^*P* < 0.001 compared with the control group. ^*^P < 0.05, ^**^P < 0.01, and ^***^P < 0.001 compared with the DNP group

The STZ injection induced hyperglycemia in rats within 1 week and the hyperglycemic condition was maintained throughout the experiment (STZ treatment: *F*_1,14_ = 427.30, *P* < 0.0001; time: *F*_2,42_ = 23.46, *P* < 0.0001; interaction: *F*_2,47_ = 24.40, *P* < 0.0001) (Fig. 2a). STZ-treated rats exhibited a lower body weight compared with the rats in the control group (STZ treatment: *F*_1,14_ = 55.08, *P* < 0.0001; time: *F*_6,98_ = 56.06, *P* < 0.0001; interaction: *F*_6,111_ = 37.94, *P* < 0.0001) (Fig. 2b). In addition, all STZ-treated rats displayed increased water intake and polyuria. Based on these features, the STZ injection induced sustained diabetes in rats. The treatment with either ammoxetine or duloxetine did not affect the blood glucose concentration or body weight in diabetic rats.

Compared with the control rats, mechanical allodynia was consistently present in diabetic rats 1–6 weeks after the STZ injection, which indicated DNP (STZ treatment: *F*_1,14_ = 1.060, *P* < 0.0001; time: *F*_6,98_ = 16.03, *P* < 0.0001; interaction: *F*_6,111_ = 11.68, *P* < 0.0001). Drugs were administrated for 4 weeks after the STZ injection. According to the two-way repeated ANOVA, both the drug treatment and the time affected the response of the diabetic rats in the Von Frey hair test (drug: *F*_4,35_ = 3.76, *P* < 0.05; time: *F*_6,245_ = 15.52, *P* < 0.0001; interaction: *F*_24,279_ = 1.44, *P* > 0.05). The Bonferroni post hoc tests revealed that chronic ammoxetine (10 mg/kg) and duloxetine (10 mg/kg) treatments significantly attenuated mechanical allodynia in diabetic rats. Moreover, 5 mg/kg ammoxetine was efficacious after 4 weeks of treatment (Fig. 2c).

### Ammoxetine increased the locomotor activity of diabetic rats

**Fig. 3:**
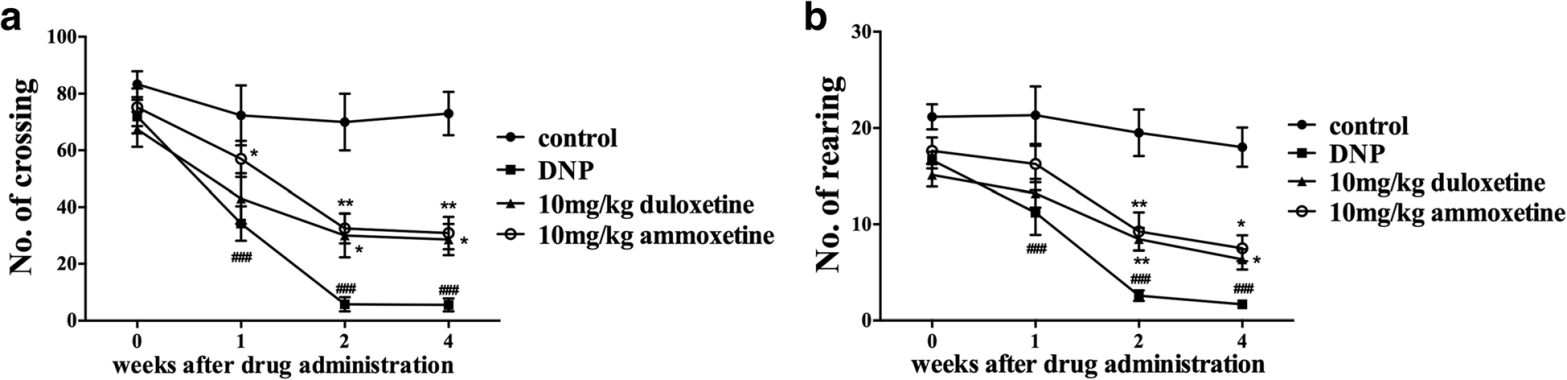
Effects of ammoxetine on the locomotor activity of DNP rats in the open field test. **a** Number of crossings. **b** Rearing in diabetic rats (*n* = 8). ^*^P < 0.05 and ^**^P < 0.01 compared with the DNP group

Based on the results from the open field test, the numbers of crossings and rearings decreased in diabetic rats beginning at 1 week after drug administration (equivalent to 3 weeks after STZ injection) compared with control rats (number of crossings: STZ treatment: *F*_1,12_ = 77.71, *P* < 0.0001; time: *F*_3,36_ = 25.30, *P* < 0.0001; interaction: *F*_3,42_ = 7.88, *P* < 0.0001; number of rearings: STZ treatment: *F*_1,12_ = 82.32, *P* < 0.001; time: *F*_3,36_ = 12.36, *P* < 0.0001; interaction: *F*_3,42_ = 5.94, *P* < 0.0001) (Fig. 3a, b). The highest doses of ammoxetine and duloxetine were examined in the open field test. According to the two-way repeated ANOVA, both the drug treatment and the time affected the numbers of crossings and rearings (number of crossings: drug treatment: *F*_2,21_ = 5.12, *P* < 0.05; time: *F*_3,84_ = 71.28, *P* < 0.0001; interaction: *F*_6,95_ = 1.99, *P* > 0.05; number of rearings: *F*_2,21_ = 6.15, *P* < 0.01; time: *F*_3,84_ = 48.25, *P* < 0.0001; interaction: *F*_6,95_ = 1.79, *P* > 0.05). The Bonferroni post hoc tests revealed that the administration of ammoxetine (10 mg/kg) significantly increased the numbers of crossings (*P* < 0.05 for weeks 1, 2, and 4) and rearings (*P* < 0.01 for week 2 and *P* < 0.05 for week 4). Duloxetine (10 mg/kg) significantly increased the numbers of crossings (*P* < 0.05 for weeks 2 and 4) and rearings (*P* < 0.05 for weeks 2 and 4).

### Effects of ammoxetine on the activation of microglia and astrocytes in the spinal cord of diabetic rats

**Fig. 4:**
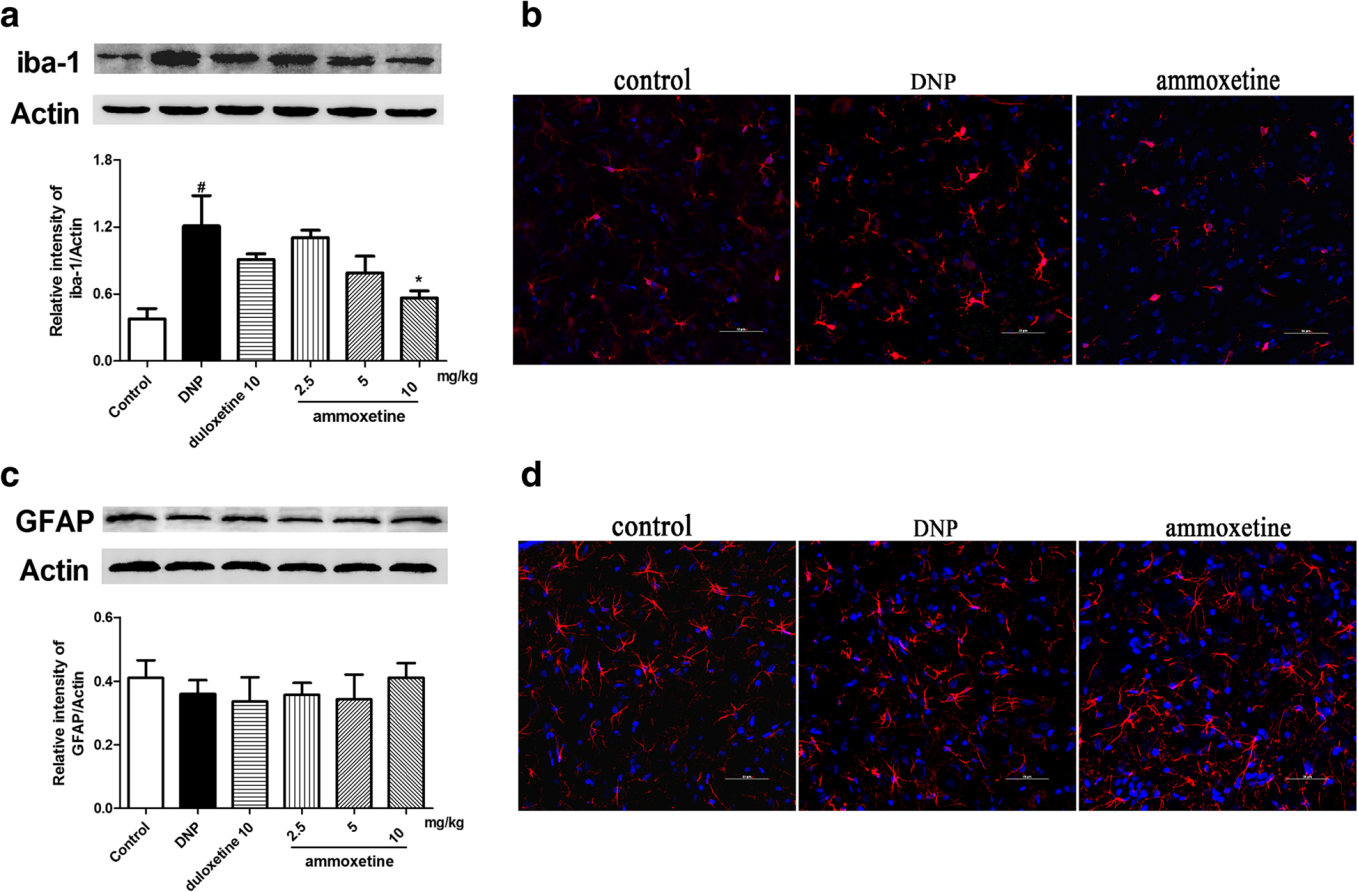
Effects of ammoxetine on the activation of microglia and astrocytes in the spinal cord of DNP rats. **a** Representative protein bands of IBA-1, the relative optical density was normalized to β-actin. **b** Images of IBA-1 labeled microglia on the lumbar spinal cord sections. **c** Representative protein bands of GFAP, the relative optical density was normalized to β-actin. **d** Images of GFAP labeled astrocytes on the lumbar spinal cord sections. ^#^*P* < 0.05 and ^##^*P* < 0.01 compared with the control group. ^*^P < 0.05 compared with the DNP group

Because the glial cells play important roles in pain hypersensitivity and multiple monoamine reuptake inhibitors have been shown to inhibit neuroinflammation, we sought to examine whether the observed anti-allodynic effect of ammoxetine in diabetic rats could be via inhibiting the activation of spinal microglia and/or astrocytes. As shown in Fig. 4, the protein level of the microglial marker IBA-1 was significantly increased in the spinal cord of diabetic rats compared with the control group of rats. The administration of ammoxetine (10 mg/kg) for 4 weeks significantly downregulated the protein level of the IBA-1 (Fig. 4a). The immunofluorescence on the lumbar spinal cord sections showed that the activation of microglia was evidently increased in diabetic rats. Treatment with ammoxetine (10 mg/kg) attenuated the upregulation of IBA-1 in diabetic rats (Fig. 4b). The protein level of the astrocyte marker glial fibrillary acidic protein (GFAP) was not significantly different between diabetic rats and the control group of rats (Fig. 4c). The data indicates that the neuropathic in the diabetic rats in the current stage might be unrelated to the activation of astrocytes in the spinal cord, which was further supported by the immunostaining of GFAP (Fig. 4d).

### Ammoxetine decreased the TNF-α, IL-1β, and IL-6 production in the spinal cord of diabetic rats

**Fig. 5:**
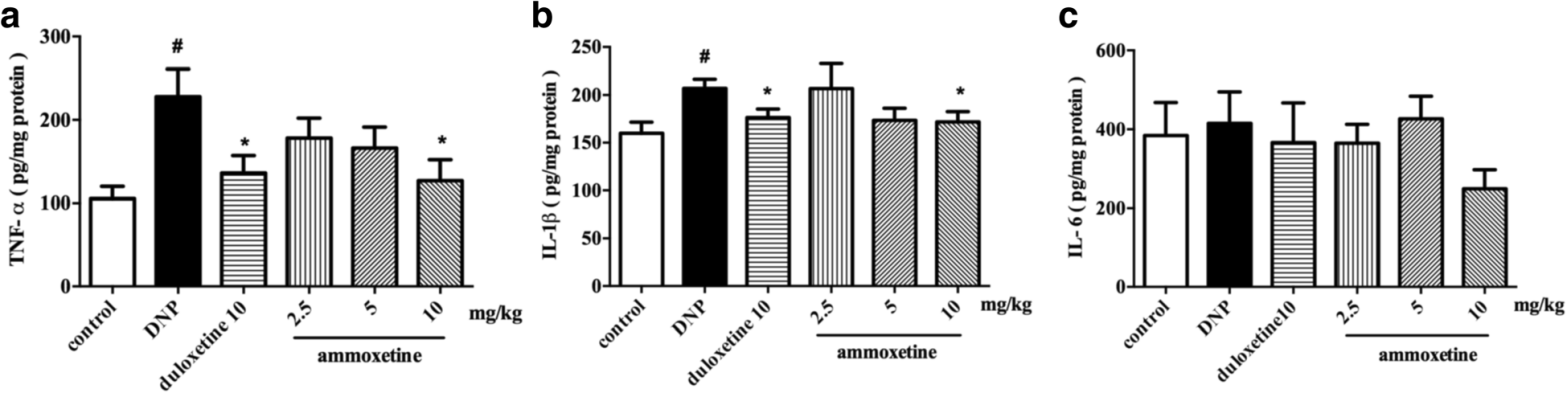
Effects of ammoxetine on pro-inflammatory cytokine production in DNP rats. Levels of **a** TNF-α, **b** IL-1β, and **c** IL-6 in the spinal cord of DNP rats that had been treated with drugs for 4 weeks were quantified using ELISAs (*n* = 6). ^#^*P* < 0.05 compared with the control group. ^*^P < 0.05 compared with the DNP group

Microglia generally respond to injury by releasing pro-inflammatory cytokines. We investigated the effect of ammoxetine on the production of inflammatory mediators in the rat model of diabetic neuropathic pain. The levels of TNF-α (*P* < 0.05) and IL-1β (*P* < 0.01), but not IL-6, were significantly increased in the spinal cord of diabetic rats compared with the control group of rats. Oral administration of 10 mg/kg ammoxetine or duloxetine for 4 consecutive weeks significantly decreased the TNF-α and IL-1β levels (Fig. 5a–c).

### Effects of ammoxetine on MAPK and NF-κB/P65 activation in the spinal cord of diabetic rats

**Fig. 6:**
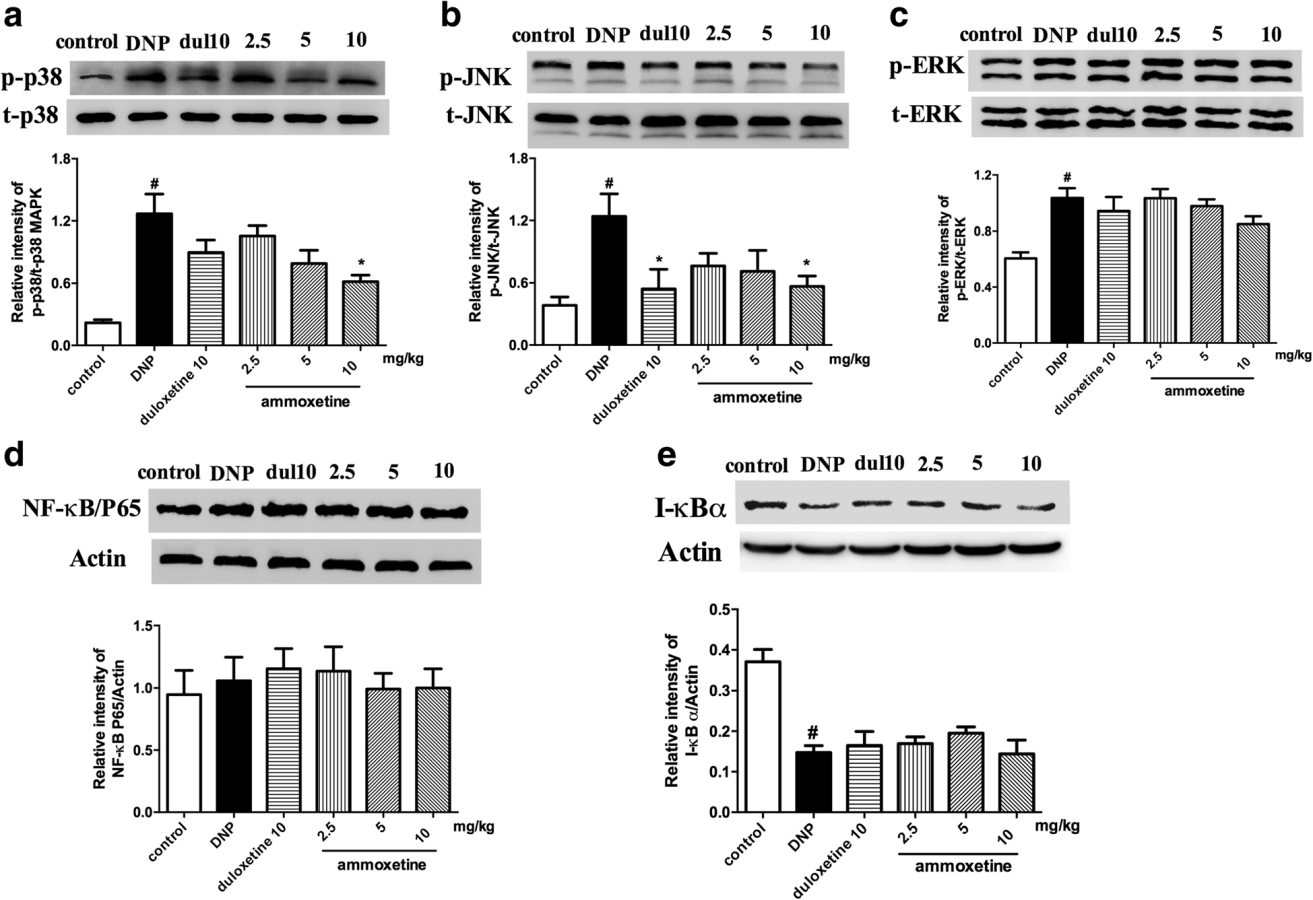
Effects of ammoxetine on the phosphorylation of JNK and ERK in the spinal cord of DNP rats. The protein levels of phosphorylated of **a** p38, **b** JNK, and **c** ERKin each group were normalized to the relative intensity of total-p38, JNK, and ERK. The levels of **d** NF-κB/P65 and **e** IκB⍺ was normalized to the relative intensity of β-actin (*n* = 3). ^#^*P* < 0.05 compared with the control group. ^*^P < 0.05 compared with the DNP group

Reports show that the protein levels of p-p38, p-JNK, and p-ERK increase in the spinal cord in multiple rat models of neuropathic pain. Therefore, we investigated whether ammoxetine is able to modulate the activation of MAPK in diabetic rats. We found that the protein levels of p-p38, p-ERK, and p-JNK were significantly increased in the spinal cord of diabetic rats compared with the control group of rats. The 4 week ammoxetine (10 mg/kg) treatment significantly inhibited the increase in the protein levels of the p-p38 and p-JNK, but not the p-ERK. Duloxetine significantly decreased the p-p38 level in diabetic rats (Fig. 6a–c).

NF-κB has been shown to be an important upstream modulator for pro-inflammatory cytokines in microglia. Moreover, previous studies indicate that the spinal NF-κB/p65 plays a critical role in the initiation and development of peripheral inflammation hyperalgesia. In the present study, we examine whether the pain-relieving effect ammoxetine in diabetic rats is related to activation of NF-κB. The data shows that no significant difference in the protein levels of the NF-κB/P65 was detected between diabetic rats and control rats (Fig. 6d). The degradation of inhibitor of NF-κB (IκBα) proteins indicating the activation of NF-κB. The protein level of IκB⍺ was significantly decreased in the spinal cord of diabetic rats compared with the control group of rats. While ammoxetine did not reverse the degradation of IκBα in diabetic rats (Fig. 6e).

### Effects of ammoxetine on the activation of BV-2 cells induced by LPS

**Fig. 7:**
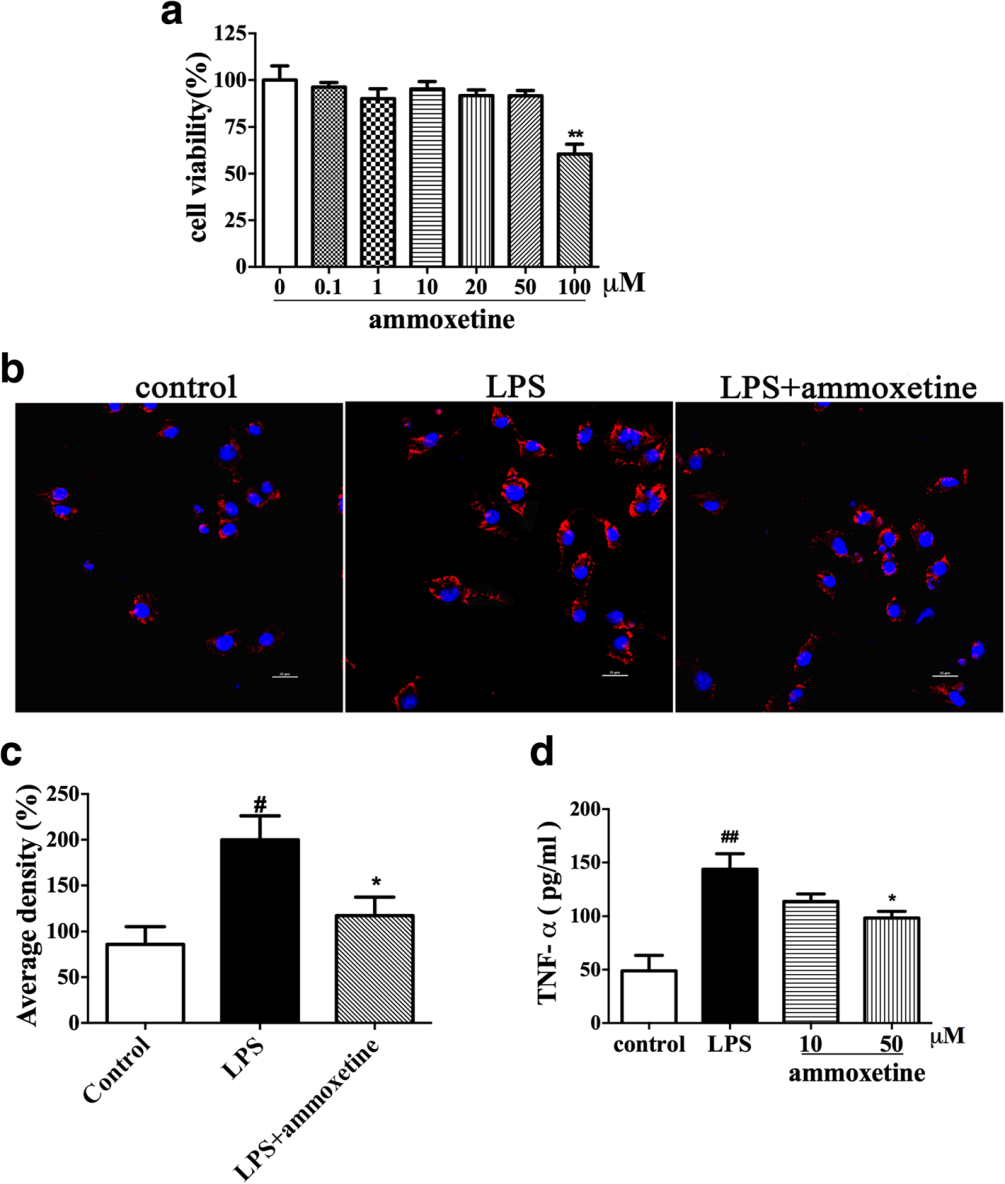
Effects of ammoxetine on the activation of BV-2 cells induced by LPS. **a** Cell viability after treated with various concentration of ammoxetine for 24 h. **b** Representative images of IBA-1 stained BV-2 microglial cells 24 h after LPS and/or ammoxetine incubation. **c** Quantification of average intensity in 3 independent images. **d** TNF-α production in the culture medium of BV-2 cells after treated with LPS or ammoxetine (*n* = 3). ^#^*P* < 0.05 compared with the control group. ^*^P < 0.05 compared with the LPS-treated group

To test a direct effect of ammoxetine on microglial activation, we sought to examine the effect of ammoxetine on the activation of BV-2 cells induced by LPS. Firstly, MTT assay was performed to determine its cytotoxicity to BV2 cells after 24 h incubation with different concentrations of ammoxetine. Cell viability following treatment with ammoxetine at 0.1, 1, 10, 20, and 50 μM was not significantly different from the control. Exposure of BV-2 cells to ammoxetine at 100 μM resulted in significantly fewer viable cells as compared to cells in the control (Fig. 7a). Then, the immunofluorescence analysis showed that LPS induced significant increase in the expression of IBA-1 and ammoxetine (50 μM) attenuated the upregulation of IBA-1 in BV-2 cells (Fig. 7b, c). We also assessed the effect of ammoxetine on the production of LPS-induced pro-inflammatory cytokines. The result shows that stimulation of BV-2 cells with LPS led to a significant increase in the TNF-α production. Treatment with ammoxetine significantly inhibited TNF-α production in BV-2 cells in a dose-dependent manner (Fig. 7d).

### Effects of ammoxetine on the activation of MAPKs and NF-κB in cultured microglia

**Fig. 8:**
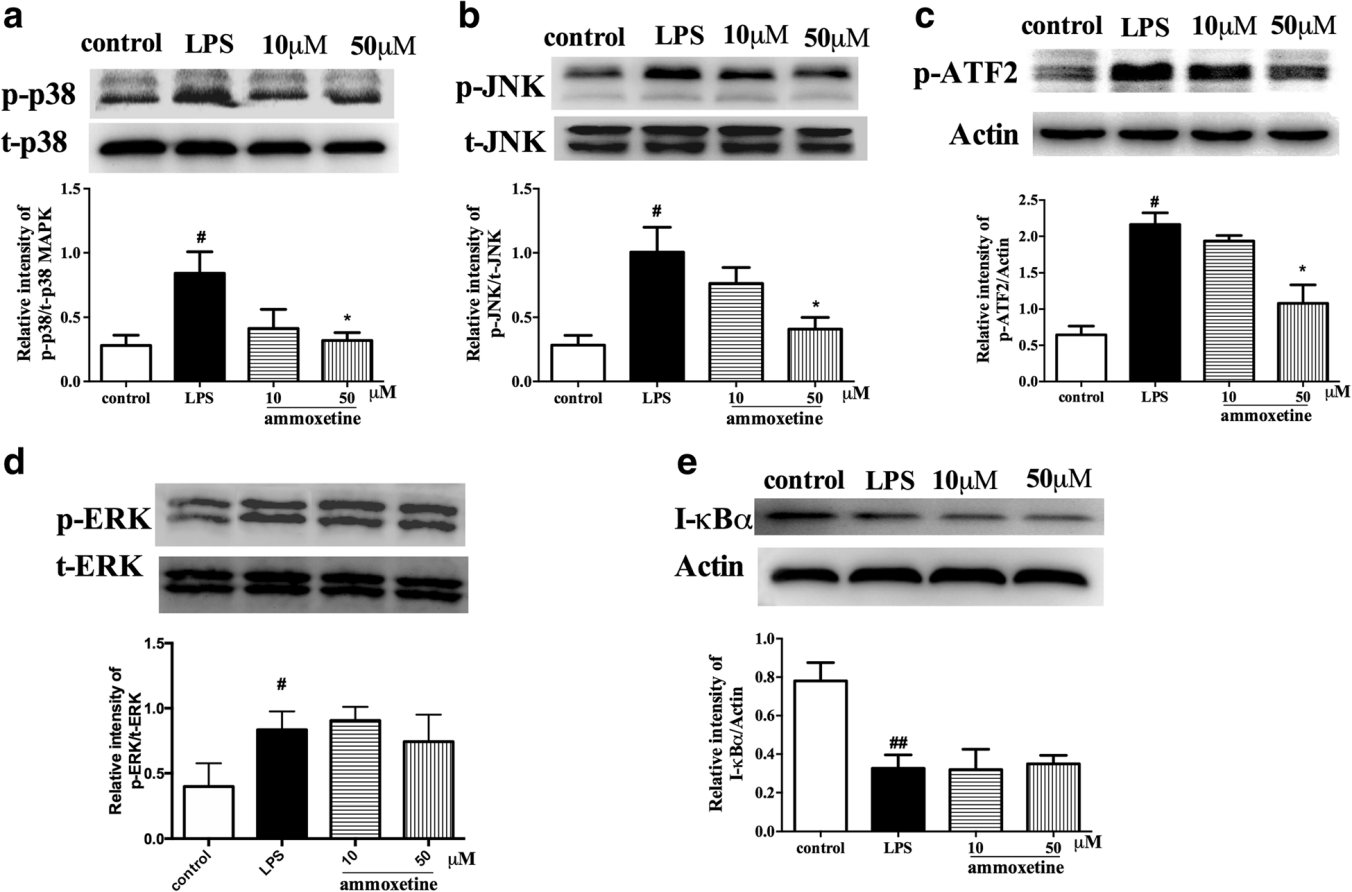
Effects of ammoxetine on the activation of MAPKs and NF-κB in cultured microglia. The protein levels of phosphorylated of **a** p38, **b** JNK, and **d** ERK were normalized to the relative intensity of total-p38, JNK, and ERK. The levels of p-ATF-2 (**c**) and IκBα (**e**) was normalized to the relative intensity of β-actin (*n* = 3). ^#^*P* < 0.05 compared with the control group. ^*^P < 0.05 compared with the the LPS-treated group

We further examined whether the repressive effect of ammoxetine on the release of pro-inflammatory cytokines occurred via MAPK and NF-κB in vitro. As shown in Fig. 8, ammoxetine inhibited the LPS-induced phosphorylation of p38 and JNK, which was further evidenced by the inhibition effect on the expression of p-ATF2 (activating transcription factor), a downstream transcription factor of MAPK (Fig. 8a–c). Ammoxetine did not reduce the LPS-induced phosphorylation level of ERK (Fig. 8d). LPS treatment induced markedly degradation of IκBα, which indicates the activation of NF-κB pathway. Ammoxetine did not reverse the LPS-induced degradation of IκBα in BV-2 cells. These findings indicate that the anti-inflammatory effect of ammoxetine may be related to inhibiting the activation of p38 and JNK MAPK.

## Discussion

The present study is the first to report that ammoxetine, a novel SNRI, presents chronic analgesic and depression ameliorative effects in the rat model of STZ-induced DNP. We also observed an increase in the levels of the inflammatory cytokines, including TNF-α, IL-1β, in the spinal cord of diabetic rats, and ammoxetine decreased the levels of TNF-α and IL-1β. In addition, ammoxetine-induced reduction in the levels of inflammatory cytokines is mediated by inhibiting the action of microglia and the phosphorylation of p38 and JNK, but is not associated with astrocytes, ERK, or NF-κB.

Rats injected with STZ displayed sensory dysfunction, such as tactile allodynia and mechanical and thermal hyperalgesia, which is the most commonly used animal model to investigate the mechanisms of DNP and evaluate potential therapeutic drugs [28, 30]. The results in our study are consistent with previous studies showing that hyperglycemia was induced 4 days after STZ injection and persisted for more than 6 weeks. Mechanical allodynia occurred 1 week after the STZ injection and persisted for more than 5 weeks [28].

According to clinical studies, the SNRI duloxetine produces direct analgesic effects, significantly reduces the doses of other required analgesic drugs in patients with DNP, and displays good tolerance and safety [31]. Duloxetine does not affect the patient’s blood glucose or blood lipid indicator levels and does not cause an abnormal electrocardiogram [32, 33]. Consistently, both ammoxetine and duloxetine chronically relieved mechanical allodynia, but did not affect the blood glucose levels and body weight in the diabetic rats during the experimental period in the present study. Meanwhile, DNP often causes sleep disturbances, anxiety, and depression [34]. These complications strongly influence the patient’s ability to function with decreased motility. In addition to the pain-relieving effect, ammoxetine simultaneously increased the locomotor activity in chronically treated DNP rats, which may indicate a mood-improving effect. We have reported similar results of ammoxetine in chronic mild stress model of depression in rat [24]. To our knowledge, these data are the first to show an effect of antidepressants on improving motility and mood in animal model of DNP. Thus, ammoxetine may have potential clinical value similar to duloxetine.

The pathogenesis of DNP is not completely understood. Despite the consensus that sustained hyperglycemia represents an important causative factor by inducing metabolic disorders, such as polyol pathway hyperactivity [35], oxidative stress [36], and microvascular changes [37], researchers have not determined why some diabetic patients develop the painful symptoms and others do not. The pain intensity does not depend on neuropathy and can occur even in the absence of nerve injuries [3]. Other theories have been proposed to explain DNP, including metabolic and autoimmune disorders accompanied by glial cell activation, sodium and calcium channel-dependent sprouting, central sensitization, and changes in brain plasticity [2].

Antidepressants, particularly duloxetine, are generally considered drugs that treat DNP symptoms, while the precise mechanism of pain relief is not clear. Although enhancements in both 5-HT and NE transmission in the descending pain control pathways have been regarded as plausible explanation [38]. However, in diabetic rats, studies on the levels of neurotransmitters in the endogenous pain control system have reported inconsistent results. Some studies observed reduced norepinephrine release in the spinal cord of diabetic rats, whereas others described opposite findings [3, 39]. The study by Morgado et al. [17] reported increased numbers of serotoninergic and noradrenergic neurons in key brainstem centers for descending pain control and increased levels of 5-HT and norepinephrine in the spinal cord of subjects with diabetes. Thus, other mechanisms might account for the DNP-relieving effects of these drugs.

Glial cells are known to play an important role in the pathogenesis of many diseases of the nervous system, including chronic pain states and mental disorders such as depression. Activated microglia and astrocytes in the spinal cord play important roles in the initiation and maintenance of traumatic neuropathic pain [40]. In the present study using an STZ-induced diabetic model, extensive microglial activation was detected 6 weeks after hyperglycemia induction, whereas astrocyte activation was not observed. Similar findings have been observed in STZ-induced diabetic rats, with extensive activation of microglia in the dorsal horn observed for 4 weeks [20] and even for 8 months [41]. As shown in the study by Cheng et al., microglial activation persisted for 6 months in the dorsal horn of STZ-induced diabetic rats, whereas reduced astrocyte activation was observed for 2 months and then returned to normal levels [42]. There is no consistent conclusion on the role of astrocytes in diabetic neuropathic pain. Studies by Tsuda and Wodarski concluded that no activation of astrocytes in STZ-treated diabetic rats [20]. While Afsari ZH et al. reported that diabetes is associated with a reduction in glial fibrillary acidic protein immunoreactive astrocytes in the spinal cord [43]. Conversely, Dauch JR et al. showed that neuron-astrocyte signaling network in the spinal cord dorsal horn mediates painful neuropathy of type 2 diabetes [44]. The study of Liao YH et al. found that spinal astrocytic activation contributes to mechanical allodynia in a mouse model of type 2 diabetes [45]. It seems that there exist differences of the activation of astrocyte between type 1 and type 2 diabetes. More research work is needed to conclude this issue.

We are the first to show that ammoxetine, a SNRI, reduced microglial activation and the production of the inflammatory cytokines TNF-α and IL-1β in the spinal cord of rats with DNP. The anti-inflammatory effects of monoamine reuptake inhibitors have been suggested to be involved in their therapeutic effectiveness for depression and traumatic neuropathic pain [46–48], but rarely for DNP. The first evidence for the anti-inflammatory effects of antidepressants was reported in 1993 in a study showing that the SSRI fluoxetine and citalopram inhibited the proliferation of mitogen-activated lymphocytes [49]. Moreover, they found that different compounds exhibited unequal anti-inflammatory efficacy, with fluoxetine exhibiting a stronger efficacy than citalopram. The same team later found that citalopram also significantly inhibited the release of TNF-α and IL-1β from activated human mononuclear cells [50]. Antidepressants have recently been shown to alter the inflammatory effects of microglia. As shown in the study by Bielecka et al. [51], reversible monoamine oxidase inhibitors (MAOIs) dose-dependently inhibited the release of TNF-α and IL-1β from mixed cultures of glial cells stimulated with LPS. The SNRI venlafaxine displayed anti-inflammatory activity in mixed cultures of glial cells [52]. Regarding the pain-relieving mechanisms, amitriptyline, milnacipran, and fluoxetine reduced microglial activation when relieving neuropathic pain in a chronic constriction injury (CCI) model [53]. According to Zhu et al. [54], the administration of mirtazapine for 14 days significantly relieved neuropathic pain in rats with spinal nerve ligation and reduced TNF-α and IL-1β release and astrocyte activation in the hippocampus; this effect was blocked by 5-HT and NE antagonists. Nortriptyline and venlafaxine were reported to reduce nerve injury-induced TNF-α release by increasing NE levels and activating non-neuronal β_2_-ARs [55]. Collectively, further investigations are required to determine whether the inhibitory effects on glial cell activation and the release of inflammatory cytokines are due to direct anti-inflammatory effects of the drugs or secondary effects on 5-HT and NE and relieving pain. IL-6 has dual roles in brain injury and disease. It is produced during reactive astrogliosis in response to neuronal damage, acting as a neurotrophin that promotes neuronal survival. Elevated levels of IL-6 have also been adversely associated with several brain diseases [56, 57]. In the present study, the level of IL-6 did not decrease in the spinal cord of diabetic rats, which may be related to the lack of astrocyte activation.

The intracellular signal transduction pathways and transduction molecules such as MAPKs play an important role in the release of inflammatory factors. As shown in the study by Daulhac et al. [58], the neuropathy observed in hyperalgesic diabetic rats 3 weeks after the STZ injection is associated with the increased phosphorylation of p38, JNK, and ERK in the spinal and dorsal root ganglia. All three phosphorylated MAPKs are co-expressed in spinal neurons and microglia, but not in astrocytes. In addition, inhibitors of p38 and JNK relieve the mechanical hyperalgesia in diabetic rats by inhibiting the phosphorylation of these kinases. In the present study, ammoxetine significantly decreased the levels of phosphorylated p38 and JNK in the spinal cord of rats with DNP, suggesting that the inhibition of p38 and JNK activation may be involved in the effect of ammoxetine on reducing microglial activation and the inflammatory response. The regulatory effects of antidepressants on MAPKs have been reported in previous studies. In vitro studies using LPS-stimulated microglial activation showed that fluoxetine inhibited p38 phosphorylation and paroxetine inhibited JNK and ERK phosphorylation, which may be one of the mechanisms underlying their anti-inflammatory activities [59]. Amitriptyline was shown to increase intercellular connexin expression by modulating the p38 pathway [60]. Antidepressants have been reported to increase ERK phosphorylation in stress-induced animal models for depression, which regarded as one of the mechanisms underlying their therapeutic effects for depression [61]. In response to nerve injury, phosphorylation of the ERK pathway promotes the expression of inflammatory cytokines in microglia [55]. In this study, ammoxetine did not inhibit ERK phosphorylation in rats with DNP, indicating that antidepressants exert disparate regulatory effects on ERK phosphorylation under different pathological conditions. Besides, we have not ruled the possibility that the inconsistency might be dependent on the different researched tissues of the spinal cord and brain.

We conducted in vitro experiments by using the model of LPS-induced activation of BV-2 microglia to investigate the direct effect of ammoxetine on the activation of microglia. The results showed that LPS incubation induced activation of microglia and the phosphorylation of JNK, ammoxetine directly inhibited the activation of microglia and the release of TNF-α. Ammoxetine reduced the phosphorylation of p38 and JNK in vitro and decreased the level of p-ATF2. ATF2 is the downstream molecules of the MAPK signal pathway and can be phosphorylated and activated by p-p38MAPK and p-JNK. The results indicate that the anti-inflammatory of ammoxetine may be contributed to inhibiting the p38MAPK and JNK signaling.

The limitation of our study is that we cannot exclude the primary target responsible for the inhibitory effect of ammoxetine on microglial cell activation and the release of inflammatory cytokines. Further studies are required to confirm the time course of the increase/activation and the observable inhibition, whether these findings are related to descending inhibitory pathway.

## Conclusions

In conclusion, chronic treatment with ammoxetine, a potent balanced 5-HT and NE reuptake inhibitor, resulted in a sustained analgesic effect and improved depressive-like behaviors in STZ-induced diabetic rats. Moreover, the data highlight a role for the inhibition of microglial activation and the production of inflammatory cytokines in the anti-allodynic mechanism of ammoxetine in DNP. Furthermore, increased phosphorylation of intracellular signaling molecules, including p38, JNK, and ERK, confirms functional activation of microglia and the neuro-immune response in DNP rats. The inhibitory effect of ammoxetine on p38 and JNK phosphorylation may represent the signal transduction mechanism underlying its analgesic effect on this model.

## Abbreviations

5-HT: Serotonin
CCI: Chronic constriction injury
DNP: Diabetic neuropathic pain
ERK: Extracellular signal-regulated kinase
IL-1β: Interleukin-1β
JNK: c-Jun N-terminal kinase
MAOIs: Monoamine oxidase inhibitors
MAPKs: Mitogen-activated protein kinases
NE: Norepinephrine
NF- κB: Nuclear factor κB
PWT: Paw withdrawal threshold
RVM: Rostral ventromedial medullary
SNRIs: Serotonin and norepinephrine reuptake inhibitors
STZ: Streptozocin
TCAs: Tricyclic antidepressants
TNF-α: Tumor necrosis factor-α
